# Contributions of Astrocyte and Neuronal Volume to CA1 Neuron Excitability Changes in Elevated Extracellular Potassium

**DOI:** 10.3389/fncel.2022.930384

**Published:** 2022-07-22

**Authors:** Erin Walch, Alexander Bilas, Valine Bebawy, Angelina Lam, Thomas R. Murphy, Sandhya Sriram, Todd A. Fiacco

**Affiliations:** ^1^Division of Biomedical Sciences, School of Medicine, University of California, Riverside, Riverside, CA, United States; ^2^Center for Glial-Neuronal Interactions, University of California, Riverside, Riverside, CA, United States; ^3^Department of Molecular, Cell and Systems Biology, University of California, Riverside, Riverside, CA, United States; ^4^Interdepartmental Graduate Program in Neuroscience, University of California, Riverside, Riverside, CA, United States; ^5^Undergraduate Major in Biology, University of California, Riverside, Riverside, CA, United States

**Keywords:** cell swelling, extrasynaptic, NMDA, slow inward currents, epilepsy, secretagogue, glutamate, extracellular space

## Abstract

Rapid increases in cell volume reduce the size of the extracellular space (ECS) and are associated with elevated brain tissue excitability. We recently demonstrated that astrocytes, but not neurons, rapidly swell in elevated extracellular potassium (^∧^[K^+^]_*o*_) up to 26 mM. However, effects of acute astrocyte volume fluctuations on neuronal excitability in ^∧^[K^+^]_*o*_ have been difficult to evaluate due to direct effects on neuronal membrane potential and generation of action potentials. Here we set out to isolate volume-specific effects occurring in ^∧^[K^+^]_*o*_ on CA1 pyramidal neurons in acute hippocampal slices by manipulating cell volume while recording neuronal glutamate currents in 10.5 mM [K^+^]_*o*_ + tetrodotoxin (TTX) to prevent neuronal firing. Elevating [K^+^]_*o*_ to 10.5 mM induced astrocyte swelling and produced significant increases in neuronal excitability in the form of mixed α-amino-3-hydroxy-5-methyl-4-isoxazolepropionic acid (AMPA)/N-methyl-D-aspartate (NMDA) receptor mEPSCs and NMDA receptor-dependent slow inward currents (SICs). Application of hyperosmolar artificial cerebrospinal fluid (ACSF) by addition of mannitol in the continued presence of 10.5 mM K^+^ forced shrinking of astrocytes and to a lesser extent neurons, which resisted swelling in ^∧^[K^+^]_*o*_. Cell shrinking and dilation of the ECS significantly dampened neuronal excitability in 10.5 mM K^+^. Subsequent removal of mannitol amplified effects on neuronal excitability and nearly doubled the volume increase in astrocytes, presumably due to continued glial uptake of K^+^ while mannitol was present. Slower, larger amplitude events mainly driven by NMDA receptors were abolished by mannitol-induced expansion of the ECS. Collectively, our findings suggest that cell volume regulation of the ECS in elevated [K^+^]_*o*_ is driven predominantly by astrocytes, and that cell volume effects on neuronal excitability can be effectively isolated in elevated [K^+^]_*o*_ conditions.

## Introduction

Astrocytes play a significant role in maintaining ion and neurotransmitter concentrations in the extracellular space. This includes uptake of glutamate and potassium that is released during synaptic transmission. Potassium influx into astrocytes during neuronal synaptic activity is redistributed through the glial syncytium to areas of lower potassium concentration, a process known as “spatial buffering” (Walz, 2000; Kofuji and Newman, 2004). Potassium movement into astrocytes associated with K^+^ spatial buffering generates an osmotic gradient coupled to movement of water into the cell, leading to transient or prolonged fluxes in cellular volume (Pasantes-Morales and Schousboe, 1989; Walz, 1992; MacVicar et al., 2002; Risher et al., 2009; Walch et al., 2020).

Cellular swelling tied to ion and water influx results in a transient decrease in the volume of the extracellular space, positively influencing neuronal excitability (Fiacco et al., 2007; Lauderdale et al., 2015; Yang et al., 2019). Dramatic reduction of the extracellular space (ECS) also occurs prior to the generation of ictal (or seizure-like) discharges (Traynelis and Dingledine, 1989; Kilb et al., 2006; Shahar et al., 2009). In 8.5 mM [K^+^]_*o*_, neurons generate synchronous ictal bursting activity that requires NMDA receptor activation and reduction of the ECS (Traynelis and Dingledine, 1988). In these conditions, although direct cell volume measurements were not taken, it was speculated that the reduction of the ECS was likely due to astrocyte swelling tied to influx of K^+^ (Traynelis and Dingledine, 1989). Therefore, astrocyte swelling may directly influence neuronal excitability, contributing to seizure generation. However, because epileptiform activity is, by definition, synchronous neuronal bursting, it is difficult to dissociate possible influences of changes in cell volume from the direct effects of elevated extracellular potassium (^∧^[K^+^]_*o*_) on neuronal spiking.

We previously used a hypoosmolar model of acute cerebral edema to examine the effects of rapid cell swelling on neuronal activity in acute mouse hippocampal slices (Lauderdale et al., 2015; Murphy et al., 2017). Cell swelling generated a burst of NMDA receptor-mediated slow inward currents (SICs) over the course of several minutes which correlated with increased action potentials and burst firing (Lauderdale et al., 2015). However, although the hypoosmolar model had previously been reported to swell only astrocytes due to selective expression of the water channel AQP4 (Manley et al., 2000; Solenov et al., 2004; Risher et al., 2009), we later found that neurons swell to a similar extent as astrocytes (Murphy et al., 2017). Therefore, it was not possible to define a specific contribution of astrocyte swelling to the neuronal excitability increases that we observed. An advantage provided by the ^∧^[K^+^]_*o*_ model is that it swells astrocytes selectively (Walch et al., 2020), presenting an alternative cell swelling model to study effects of astrocyte swelling on neuronal excitability. In this study, we recorded excitatory postsynaptic currents (EPSCs) in CA1 pyramidal neurons while modifying extracellular solution osmolarity without altering [K^+^]_*o*_. By quantitative comparison of event frequency and event characteristics occurring in ^∧^[K^+^]_*o*_ before and during forced reduction of cell volume, a volume-dependent component of ^∧^[K^+^]_*o*_-induced neuronal excitability was isolated from hyperosmolarity-induced forced vesicular fusion, which generates fast synaptic events. Our findings suggest that astrocyte swelling in ^∧^[K^+^]_*o*_ increases neuronal excitability through the generation of large amplitude, slower extrasynaptic excitatory currents that are diminished by astrocyte volume reduction and forced expansion of the ECS.

## Materials and Methods

All experiments were performed in accordance with National Institutes of Health guidelines for the care and use of laboratory animals and approved by the Institutional Animal Care and Use Committee at the University of California, Riverside.

### Acute Hippocampal Slice Preparation

Acute hippocampal slices were prepared from C57Bl/6J mice or transgenic mice on the C57Bl6 background at 15–21 days of age as described previously (Xie et al., 2014). In some astrocyte imaging experiments, use of hippocampal slices from mGFAP-Cre;Rosa26*^lsl–tdTomato^* transgenic mice replaced C57Bl/6J mice. For experiments requiring neuronal imaging, Thy1-GFP-S or Thy1-GFP-M transgenic mice were used instead (stock #s 011070 and 007788, respectively; Jackson Laboratories, Bar Harbor, ME, United States). These mice have been repeatedly backcrossed to a C57Bl/6J background and exhibit no obvious differences in phenotype compared with wild-type mice (Feng et al., 2000).

Animals were anesthetized under isoflurane and rapidly decapitated, and brains were removed quickly into partially frozen slicing buffer bubbled with Carbogen (95% oxygen and 5% carbon dioxide). Parasagittal slices (350 μm thick) were cut using an automated vibrating blade microtome (VT1200S model; Leica Biosystems, Buffalo Grove, IL, United States) in slicing buffer and trimmed until only the hippocampus and immediately surrounding tissues remained. Slicing buffer contained (in mM): 87 NaCl, 75 sucrose, 10 glucose, 1.25 NaH_2_PO_4_, 2.5 KCl, 25 NaHCO_3_, 1.3 ascorbic acid, 0.5 CaCl_2_, 7 MgCl_2_, 2 pyruvate, and 0.1 kynurenic acid. These mini-slices were transferred to a recovery chamber containing 36°C slicing buffer solution and incubated in a 36°C water bath for 45 min. After incubation, the slicing chamber was moved to room temperature (RT) for 15 min. Slices were then transferred into another chamber containing control ACSF comprised of (in mM): 125 NaCl, 2.5 KCl, 2.5 CaCl_2_, 1.3 MgCl_2_, 1.25 NaH_2_PO_4_, 26 NaHCO_3_, and 15 glucose. Slices rested in ACSF for a minimum of 15 min at RT prior to the start of experiments.

### Electrophysiology

Following recovery, slices were transferred to a recording chamber and continuously perfused with oxygenated control ACSF at room temperature. Slices and individual CA1 pyramidal neurons were visualized on a Leica DLMFSA upright microscope, with HCX APO L20x/0.50W U-V-I and HCX APO L63x/0.90W U-V-I submersion objectives and differential interference contrast optics (Leica Microsystems, Buffalo Grove, IL, United States). Whole-cell patch clamp recordings were acquired using a MultiClamp 700B amplifier and Digidata 1550 digitizer, controlled using pCLAMP v.10.7.0.3 and MultiClamp Commander v.2.2.2.2 software (Molecular Devices, San Jose, CA, United States). Patch pipettes were pulled from thin-wall 1.5 mM borosilicate glass capillaries (World Precision Instruments) using a Narishige PC-10 vertical micropipette puller (Narishige, Tokyo, Japan). Patch pipette resistances ranged from ∼3–5 mOhm when filled with an internal solution containing the following (in mM): 140 K-gluconate, 4 MgCl_2_, 0.4 ethylene glycol-bis(β-aminoethyl ether)-N,N,N′,N′-tetraacetic acid (EGTA), 4Mg^2+^-ATP, 0.2 Na-GTP, 10 2-[4-(2-hydroxyethyl)piperazin-1-yl]ethanesulfonic acid (HEPES), and 10 phosphocreatine, pH 7.3 with KOH. CA1 pyramidal neurons were identified using differential interference contrast optics based on their location in stratum pyramidale and their characteristic morphology including apical dendrites arborizing into the hippocampal molecular layer. Upon attaining the whole-cell configuration, the cell resting V_*m*_ and R_*m*_ were recorded, and a voltage-step protocol was run to verify the presence of voltage-gated sodium and potassium currents. In voltage clamp mode, neurons were held at the chloride reversal potential of V_*m*_ = −70 mV during continuous recording of excitatory currents (I) in pA. Only a single neuronal recording was made per slice.

### Experimental Protocols and Pharmacology

Neurons were patch-clamped in control ACSF at the start of each experiment. Control ACSF contained 2.5 mM KCl as described above and had a final osmolarity of ∼300 mOsm. After initiating a continuous recording of membrane currents, solution was switched to 0 mM MgCl_2_ (Mg^2+^-free) ACSF to remove Mg^2+^ block of NMDA receptors, plus 1 μM TTX to block voltage-gated sodium channels. After 10 min of baseline recording in these conditions, extracellular KCl was raised to 10.5 mM (^∧^[K^+^]_*o*_ ACSF) for 5 min. 10.5 mM [K^+^]_*o*_ was chosen for these experiments based on our previous work assessing effects on neuronal and astrocyte volume and mechanisms of astrocyte swelling at this concentration (Walch et al., 2020), which falls within the 10–12 mM ceiling range of extracellular K^+^ that can be reached during high-frequency stimulation or epileptiform activity (Moody et al., 1974; Heinemann and Lux, 1977; Walz, 2000). NaCl was reduced by an equivalent amount in ^∧^[K^+^]_*o*_ ACSF to maintain solution osmolarity. Solution was then switched back to the baseline condition for 10 min (^∧^[K^+^]_*o*_ wash) and this sequence was repeated. A second experimental protocol was a modification of the above, except that 10.5 mM [K^+^]_*o*_ ACSF was applied and maintained for the duration of the recording, with two sequential 10 min periods in 40% hyperosmolar ^∧^[K^+^]_*o*_ ACSF (∼420 mOsm) by addition of D-mannitol with a 10 min mannitol wash period in-between. Mannitol is a membrane-impermeant solute which has been used experimentally and clinically to dilate the extracellular space by forcing cellular volume reduction (Traynelis and Dingledine, 1989; Dudek et al., 1990; Roper et al., 1992; Haglund and Hochman, 2005; Kilb et al., 2006). Additional experiments in ^∧^[K^+^]_*o*_ ACSF + mannitol as described above were performed in the presence of 10 μM NBQX (Alomone Labs) to block AMPA receptor currents (NMDA receptor isolation), or with the addition of 50 μL DL-AP5 (Abcam) to block remaining NMDA receptor currents (NMDA receptor inhibition). For all protocols, membrane potentials, membrane and series resistances, and holding currents were recorded at the end of exposure to each solution.

### Event Analysis

Voltage-clamp recordings of CA1 pyramidal neurons were analyzed for individual events in the form of currents (pA). Clampfit software (Molecular Devices) was used to extract characteristics from individual events: Peak amplitude, 10–90% rise time, time to peak and decay tau. For the purposes of this study, miniature excitatory post-synaptic currents (mEPSCs) were defined as events having a rise time <10 ms, and slow inward currents (SICs) with rise times ≥10 ms and amplitudes ≥20 pA (Angulo et al., 2004; Fellin et al., 2004). Because there were a large number of events that did not fit into either category (i.e., rise times slower than 10 ms, and amplitudes <20 pA), all events were also analyzed as a separate category in volume manipulation experiments. Generally, the kinetics of mixed mEPSCs recorded in this study were slower than the ∼3 ms rise times of mEPSCs in other studies (Zhang et al., 2005). This difference is attributed to a combination of factors, including slower intrinsic kinetics of NMDA receptor-mediated synaptic events, slice recording temperature, ACSF perfusion rate, rise time parameters (e.g., 10–90% vs. 20–80%), and use of different analysis software.

Two different methods were used to detect events. A threshold-based search was used to identify events using the following parameters: (1) negative-going event polarity; (2) event-finding trigger, −20 pA; (3) event-finding re-arm, −1e4 pA; (4) noise rejection, 1 ms; (5) pre-trigger length, 100 ms; (6) post-trigger length, 1,000 ms; and (7) tau calculated at 10% of peak. Alternatively, a template-based search was used to detect events from a user-made representative event template from each recording. Creation of an event template consisted of averaging 5–6 events, each contained within an 80 ms duration of baseline recording. This template was then used to detect all events in the recording. The parameters of the template were: (1) negative-going event polarity; (2) baseline set “in template”; (3) match threshold 5; and (4) tau calculated from 10% of peak. The template-based detection strategy was ineffective at detecting large events with slow kinetics; i.e., slow inward currents. Therefore, a final pool of events was comprised of threshold-detected events with amplitudes >20 pA and template-detected events with amplitudes <20 pA. This method prevented events from being double-counted. Average SIC traces were obtained by extracting all SIC events from each specific application section of a recording using Clampfit software. These section-based extracted traces were then averaged together to obtain an average SIC trace for each section for each recorded cell (*n* = 13). Average traces were then grouped by application period (^∧^[K^+^]_*o*_ + mannitol 1st, mannitol wash, + mannitol 2nd) and the averages in each of these groups were averaged together to produce an overall average SIC for all cells for each application period. The average SIC traces for the initial ^∧^[K^+^]_*o*_ application and the first ^∧^[K^+^]_*o*_ + mannitol co-application period were overlayed, as were the average SIC traces for mannitol wash and second ^∧^[K^+^]_*o*_ + mannitol co-application period, for the purpose of comparison.

### Imaging

Transgenic mGFAP-Cre;Rosa26*^lsl–tdTomato^* mice express fluorescent tdTomato protein in astrocytes, which allows for imaging of astrocytes in hippocampal slices without exogenous fluorochromes. For astrocyte imaging in C57Bl/6J mice, astrocytes were incubated in 36°C slicing buffer supplemented with 1 μM sulforhodamine-101 (SR-101; Sigma-Aldrich, St. Louis, MO, United States) as described previously (Schnell et al., 2015; Murphy et al., 2017). After a 45 min incubation at 36°C, slices were transferred to room temperature (RT) conditions for 10 min. Slices were subsequently transferred to a chamber containing ACSF without SR-101 and sat for an additional 10 min. Slices were subsequently transferred to another chamber of ACSF at room temperature where they remained for a minimum of 10 min before the start of the experiment. All experiments were performed at room temperature.

Throughout our experiments, enhanced green fluorescent protein (eGFP) was excited using a 488 nm argon laser and detected with a 503–548 nm bandpass filter, and SR-101 was excited with a 559 nm semiconductor laser and detected with a 624–724 nm bandpass filter using an Olympus FluoView FV1000 (FV10-ASW) confocal imaging system. The objective used was an Olympus LUMPlanFl 60x/0.90 W 1/0 water immersion objective lens. Confocal laser settings and volume imaging of neurons and astrocytes was performed as described previously (Murphy et al., 2017). Output power for both lines was kept as low as possible (1.5%) to minimize the possibility of light-induced artifacts. Briefly, z-stacks of 1 μm-thick images were collected at 1 min intervals over individual cell somata within 15 s to ensure adequate coverage of the soma and main processes while also preventing data loss caused by tissue volume changes. Stacks were collected using a 2–3.5 zoom factor, with a scan area clipped close around the soma to increase imaging speed. Cell drift in the *x*-*y*-*z* planes was compensated using quick X-Y scans and X-Y-Z adjustments as necessary between time points. Imaging experiments consisted of three steps: (1) 10-min baseline period in Mg^2+^-free ACSF, in which 2–3 z-stacks were acquired in succession at 1-min intervals and averaged to serve as the baseline comparison for later time points; (2) a 5-min application of ^∧^[K^+^]_*o*_ ACSF, during which z-stacks through the full thickness of the cell were collected each minute; and (3) a 10-min exposure period in 40% hyperosmolar ^∧^[K^+^]_*o*_ ACSF, during which an image was taken every 5 min. Steps (2) and (3) were then repeated once each, after which the slice was discarded. Only a single cell was imaged per slice. Fiji/ImageJ was used for thresholding of image stacks and subsequent volume analysis as previously described (Murphy et al., 2017). Soma area of compressed image stacks (used as a proxy for cell volume) was analyzed with cell volume changes reported as percent change from the averaged baseline.

### Statistical Analysis

Statistical analysis was performed using SPSS Statistics 24 software and Laerd Statistics methodology (Laerd Statistics, 2015). Datasets were first assessed for “extreme” outliers by inspection of SPSS boxplots and evaluation of studentized residuals. Outliers were rarely excluded from analysis due to the small sample size of our datasets. Data were assessed for violations of normality with the Shapiro–Wilk test, which determined the selection of either a parametric or non-parametric statistical test. For analysis of volume measurements, a two-way mixed analysis of variance (ANOVA) was used to check for significant interactions between the within-subject (time) and between-subjects (group/condition) factors as previously described (Walch et al., 2020). Homogeneity of variance was assessed with Levene’s test for equality of variance. Box’s test established equality of covariances, and Mauchly’s test determined sphericity of the data set. If the assumption of sphericity was violated, the Greenhouse–Geisser estimation was used to determine significance in the two-way mixed ANOVA. Following the procedure used in Walch et al. (2020), an unequal variance *t*-test (Welch’s test) was used to check for significant differences in volume change between astrocytes and neurons at each time point. For imaging experiments, each group (astrocytes or neurons) contained *N* = 8–12 cells (numbers specified in each experiment), unless otherwise noted.

For analysis of electrophysiological data, event frequency, peak amplitude, and rise time from each cell were extracted and averaged. Averages from each cell were then averaged together, yielding a sample size (N) of 9–13 cells per experiment. Cell number was used to determine standard deviation and standard error (SE) for all experiments, except cumulative probability analysis which was based on individual events (see below). Nearly all datasets violated the assumption of normality, so non-parametric tests were used to determine significance. Within-subject changes in average event parameters across two experimental conditions were assessed using the Wilcoxon Signed Rank test. Within-subject changes in event parameters across multiple experimental conditions were assessed with Friedman’s Two-Way Analysis by Ranks. Where the Friedman test indicated significant group differences, *post hoc* pairwise comparisons were performed, and *p*-values adjusted using the Bonferroni method. These tests can only be used with complete recordings, so cells with only partial recordings were excluded from these analyses. Cells with an application period that had no events were also excluded from the analyses. Cumulative probability distributions were obtained by combining an equal number of the specified parameter from each cell. The number of events included was based on the least active cell (or the cell with the fewest events). For cumulative probability analysis, differences between the distributions were analyzed with the Kolmogorov–Smirnov test. The Mann–Whitney *U* test was used to determine if there were differences in event characteristics in individual applications between electrophysiology experiments. Error bars in all graphs indicate the standard error of the mean (SEM). Significance values are listed as follows: *p < 0.05, ^**^p < 0.01, and ^***^p < 0.001.

## Results

### Neuronal Excitability Increases Recorded in ^∧^[K^+^]_*o*_ Include a Mix of AMPA/NMDA Miniature Excitatory Post-synaptic Currents and Slow Inward Currents

**FIGURE 1:**
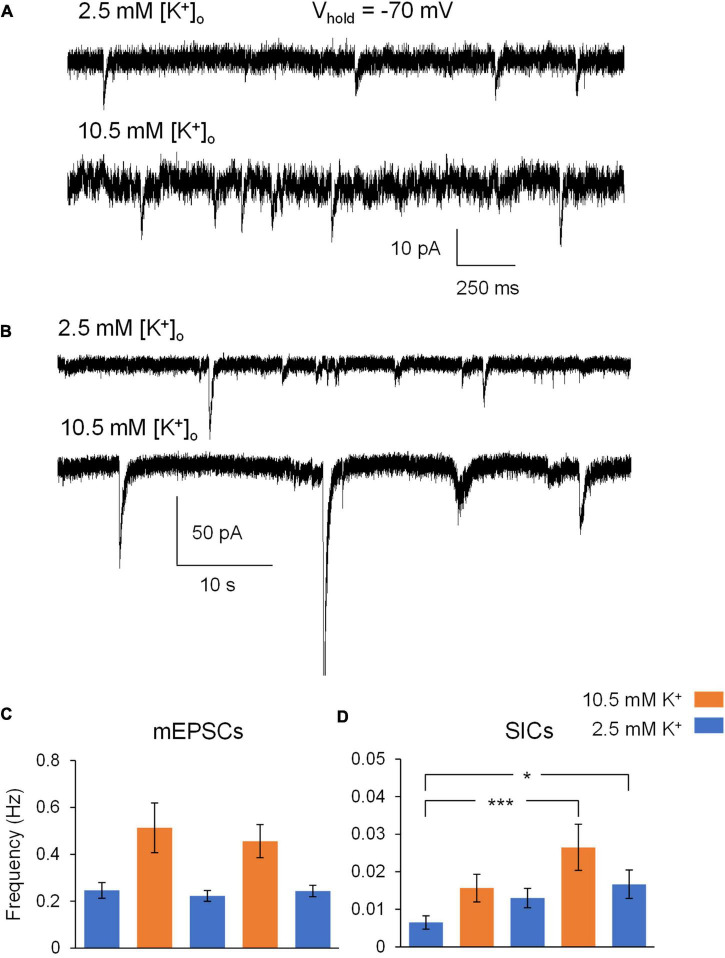
Exposure of hippocampal slices to ^∧^[K^+^]_*o*_ ACSF leads to increases in neuronal excitability. **(A)** Cropped sections of a gap-free recording of mixed AMPA/NMDA mEPSCs from a representative CA1 pyramidal neuron during whole-cell patch clamp electrophysiology (voltage clamped at –70 mV) during exposure to Mg^2+^-free ACSF (top) and Mg^2+^-free ^∧^[K^+^]_*o*_ ACSF (bottom) in 1 μM TTX. **(B)** Cropped sections of a gap-free recording of slow-inward currents from a representative CA1 pyramidal neuron during whole-cell patch clamp electrophysiology (voltage clamped at –70 mV) during exposure to Mg^2+^-free ACSF (top) and Mg^2+^-free ^∧^[K^+^]_*o*_ ACSF (bottom) in 1 μM TTX. **(C)** Exposure to ^∧^[K^+^]_*o*_ ACSF led to an increase in average mEPSC frequency during both the 1st and 2nd (5 min) applications, in comparison to the three (10 min; 0–5 min and 5–10 min) recording periods in control ACSF (*n* = 11). **(D)** Initial exposure to ^∧^[K^+^]_*o*_ ACSF led to an increase in average SIC frequency in comparison to the baseline SIC frequency in control ACSF. Increased SIC frequency persisted throughout the remainder of the experiment, despite two subsequent (10 min) periods in control ACSF. *n* = 9 neuronal recordings; 1 recording per slice. *p < 0.05 and ***p < 0.001.

We first set out to measure excitability effects of ^∧^[K^+^]_*o*_ ACSF on CA1 pyramidal neurons. The baseline concentration of extracellular potassium was 2.5 mM K^+^, while [K^+^]_*o*_ was raised to 10.5 mM in ^∧^[K^+^]_*o*_ ACSF. This concentration of [K^+^]_*o*_ is near the ceiling level that is reached during intense neuronal stimulation in intact brain tissue [reviewed in Larsen et al. (2016)]. TTX (1 μM) was included in all solutions in order to minimize direct depolarizing effects of ^∧^[K^+^]_*o*_ on action potential-driven synaptic transmission, and Mg^2+^-free ACSF was used to remove Mg^2+^ block of NMDA receptors. Application of ^∧^[K^+^]_*o*_ ACSF appeared to increase the frequency of mixed AMPA/NMDA receptor-mediated mEPSCs (Figure 1A) and slow inward currents (SICs) (Figure 1B). In 2.5 mM K^+^ ACSF, the frequency of mEPSCs was approximately ∼0.25 Hz, while ^∧^[K^+^]_*o*_ ACSF triggered mEPSC frequencies over ∼0.4 Hz in both the 1st and 2nd ^∧^[K^+^]_*o*_ applications (Figure 1C). However, these differences were not statistically significant (*p* > 0.05 for pairwise comparisons after *post hoc* correction). In comparison to mEPSCs, SIC events occurred with much less frequency overall, with the highest recorded frequencies detected near ∼0.025 Hz during the application of ^∧^[K^+^]_*o*_ ACSF (Figure 1D). Frequency of SICs increased significantly during the second application of 10.5 mM [K^+^]_*o*_ compared to the baseline period in control [K^+^]_*o*_ (*p* < 0.001), and remained elevated during subsequent periods in control ACSF (Figure 1D). There were no significant differences observed in the amplitude of mEPSCs or SICs in 10.5 mM [K^+^]_*o*_ compared to 2.5 mM [K^+^]_*o*_ (mEPSCs: 14.85 ± 0.72 pA baseline; 16.37 ± 1.42 1st ^∧^[K^+^]_*o*_; 14.40 ± 0.85 pA 1st ^∧^[K^+^]_*o*_ wash; 15.35 ± 0.93 pA 2nd ^∧^[K^+^]_*o*_; 14.63 ± 1.00 2nd ^∧^[K^+^]_*o*_ wash; *p* > 0.05 for pairwise comparisons after *post hoc* correction; SICs: 39.54 ± 4.56 pA baseline; 45.26 ± 13.07 1st ^∧^[K^+^]_*o*_; 39.51 ± 1.95 pA 1st ^∧^[K^+^]_*o*_ wash; 40.30 ± 3.86 pA 2nd ^∧^[K^+^]_*o*_; 37.44 ± 1.89 2nd ^∧^[K^+^]_*o*_ wash). In summary, the main effect of ^∧^[K^+^]_*o*_ on excitability of CA1 pyramidal neurons was an increase in the frequency of SICs in comparison to the respective baseline frequencies in 2.5 mM [K^+^]_*o*_.

### Increasing Osmolarity of ^∧^[K^+^]_*o*_ ACSF Leads to Shrinking of Astrocytes and Neurons

**FIGURE 2:**
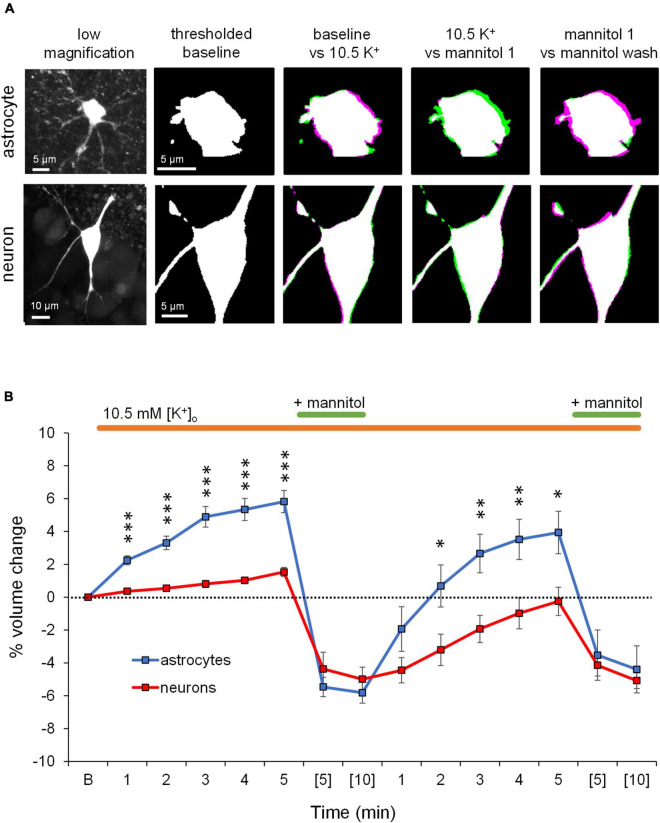
Application of mannitol in the presence of ^∧^[K^+^]_*o*_ decreases the volume of both astrocytes and neurons. **(A)** Representative confocal images of a stratum radiatum astrocyte (top) and CA1 pyramidal neuron (bottom) used in volume imaging experiments (far left). The second column shows the baseline volume of the astrocyte and neuron somata in binarized thresholded images (white). The next three columns show overlaid images of the astrocyte and neuron cell soma during each of the specified conditions. Magenta regions indicate increases in cell volume, while green regions indicate reductions in cell volume. Application of ^∧^[K^+^] ACSF triggered noticeable astrocyte volume increase compared to baseline, while neuronal volume remained relatively constant. In the continued presence of ^∧^[K^+^]_*o*_, introduction of mannitol triggered astrocyte and neuron volume decrease compared to ^∧^[K^+^]_*o*_ alone. During the washout of mannitol, astrocytes dramatically increased in volume, while neuronal volume mostly recovered to baseline. **(B)** Baseline soma volume was determined as the average soma area calculated from three z-stacks taken at minutes 8, 9, and 10 in control ACSF. Graph depicts average percent volume change from baseline (“B”) of astrocytes (blue, *n* = 11) and neurons (red, *n* = 13) during continuous exposure to ^∧^[K^+^]_*o*_ ACSF (orange bar) and two 10-min exposures to mannitol (green bar). One z-stack of the cell soma was collected every minute during exposure to ^∧^[K^+^]_*o.*_ During combined exposure to ^∧^[K^+^]_*o*_ and mannitol, a z-stack was collected at minutes (5) and (10). Note significant shrinkage of astrocyte somas by nearly 10% (*p* < 0.001) compared to approximately 3% (*p* < 0.01) by neurons during the 1st application of dual exposure to ^∧^[K^+^]_*o*_ and mannitol. Washout of mannitol resulted in a rebound of astrocyte volume to approximately 3% above baseline (*p* < 0.01), while neuron volume increased minimally. Asterisks denote significant differences between astrocyte and neuron volume at each timepoint. *p < 0.05, ^**^p < 0.01, and ^***^p < 0.001.

We previously found that neuronal volume is unperturbed by extracellular K^+^ increases ranging up to 26 mM, while astrocytes swell in a concentration-dependent manner (Walch et al., 2020). After confirming the excitability increases of pyramidal neurons exposed to ^∧^[K^+^]_*o*_ ACSF (Figure 1) and reflecting on the selective swelling of astrocytes in response to ^∧^[K^+^]_*o*_ ACSF (Walch et al., 2020), we next aimed to block or prevent astrocyte swelling in ^∧^[K^+^]_*o*_ by applying 40% hyperosmolar ACSF by addition of mannitol in the continued presence of ^∧^[K^+^]_*o*_ (Figure 2). Astrocyte volume increased in response to ^∧^[K^+^]_*o*_ as expected, while neuronal volume remained relatively constant (Figure 2). Next, in the continued presence of ^∧^[K^+^]_*o*_, 40% hyperosmolar ^∧^[K^+^]_*o*_ ACSF was applied to force volume reduction of astrocytes (Figure 2B). Despite astrocyte swelling >5% from baseline in ^∧^[K^+^]_*o*_ (*p* < 0.01), the increased extracellular osmolarity triggered a significant astrocyte volume decrease (to ∼ 6% *below* baseline; *p* < 0.001). Neurons, which don’t swell in ^∧^[K^+^]_*o*_, also shrank in hyperosmolar conditions to ∼5% below baseline volume after 5 min (*p* < 0.01) (Figure 2B). Neuronal shrinking in hyperosmolar conditions was not surprising, as we previously found that neurons readily swell in hypoosmolar conditions (Murphy et al., 2017). However here, as in our previous study (Walch et al., 2020), astrocyte shrinking was significantly greater than that of neurons in hyperosmolar conditions, perhaps due to softer viscoelastic properties of astrocytes compared to neurons (Lu et al., 2006). While astrocyte and neuron volume dropped well below baseline in hyperosmolar ACSF in ^∧^[K^+^]_*o*_, subsequent return to normosmolar ^∧^[K^+^]_*o*_ conditions (mannitol wash) over a 5 min period triggered an ∼8% change in volume (ΔV) in astrocytes with >2% overshoot above baseline (*p* < 0.01), while neuronal volume nearly recovered to baseline levels (Figure 2B). The more pronounced ΔV in astrocytes upon mannitol removal is likely because astrocytes are still pumping K^+^ into the cell (Walch et al., 2020) despite the presence of mannitol, resulting in a “rebound” volume increase when mannitol is removed. Subsequent reintroduction of mannitol for 10 min again forced volume reduction of both astrocytes and neurons, to approximately 5% below baseline (Figure 2B). The shrinking of both cell types in response to hyperosmolar ^∧^[K^+^]_*o*_ indicates that reduction of ECS volume cannot be attributed solely to astrocytes under these conditions, although the net change in volume of astrocytes is approximately double that of neurons, suggesting that astrocyte volume change is the largest contributor to expansion of the extracellular space by mannitol in ^∧^[K^+^]_*o*_.

### Hyperosmolar-Induced Cell Shrinking Isolates Volume Contribution to Neuronal Excitability in ^∧^[K^+^]_*o*_

The stark effects of mannitol on astrocyte and neuron volume in the continued presence ^∧^[K^+^]_*o*_ encouraged us to quantify changes in CA1 neuron excitability under these same conditions. Frequency, peak amplitude, and rise time of spontaneous AMPA and NMDA receptor events were recorded from voltage-clamped CA1 hippocampal neurons over a period of 50 min in either ^∧^[K^+^]_*o*_ alone or in alternating periods in ^∧^[K^+^]_*o*_ + mannitol. Events were classified as mEPSCs if their rise times were < 10 ms, or as slow inward currents (SICs) if rise times ≥10 ms and peak amplitude ≥20 pA as defined previously (Fellin et al., 2004).

**FIGURE 3:**
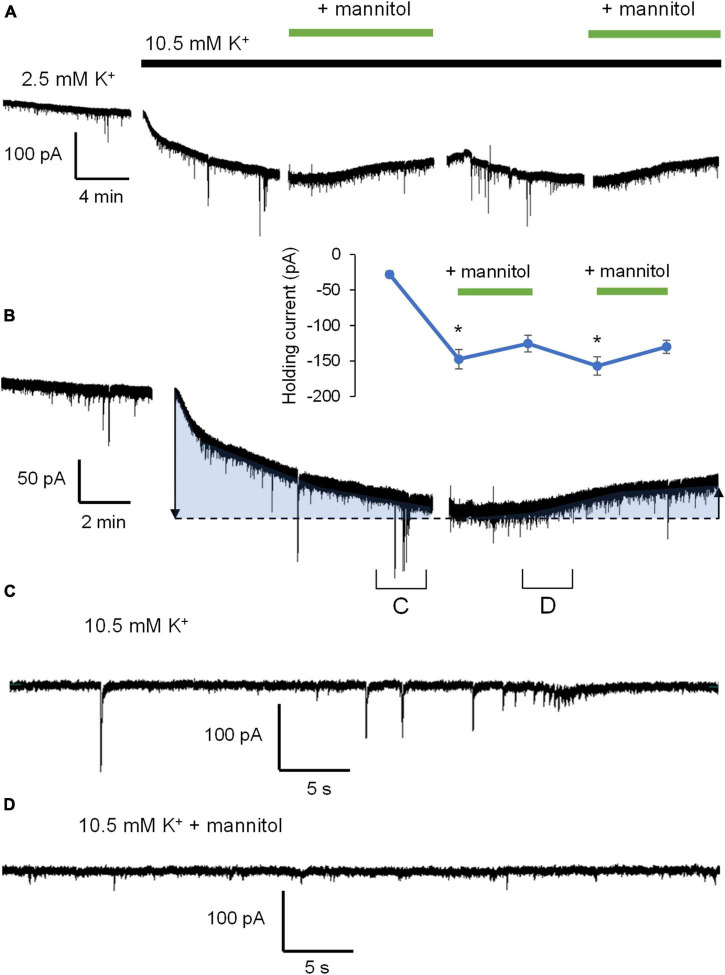
Application of hyperosmolar ^∧^[K^+^]_*o*_ ACSF triggers changes in both tonic and phasic excitatory currents. **(A)** Representative recording of a neuron voltage-clamped at –70 mV for the duration of the experiment in Mg^2+^-free ACSF + 1 μM TTX. Gaps in recordings coincide with periodic measurement membrane potential and holding current in order to reassess patch-clamp quality between alternating 10-min ACSF treatments: Control ACSF, ^∧^[K^+^]_*o*_, ^∧^[K^+^]_*o*_ + mannitol, mannitol washout, and ^∧^[K^+^]_*o*_ + mannitol (10 min ea.). **(B)** Partial section of ACSF baseline, 10-min application of ^∧^[K^+^]_*o*_, and 10 min application of ^∧^[K^+^]_*o*_ with mannitol added to show greater detail of changes in activity. Shaded sections correspond to changes in holding current indicating depolarization or hyperpolarization during each application. The inset shows the value of holding current measured at the end of each application period. **(C)** An ∼1 min section of the recording in **(B)** showing individual events during exposure to ^∧^[K^+^]_*o*_. **(D)** An ∼1 min section of the recording in **(B)** showing individual events during exposure to ^∧^[K^+^]_*o*_ with mannitol. Note the prominence of large SIC events in ^∧^[K^+^]_*o*_ and smaller mEPSC events in hyperosmolar ^∧^[K^+^]_*o*_. *p < 0.05.

**FIGURE 4:**
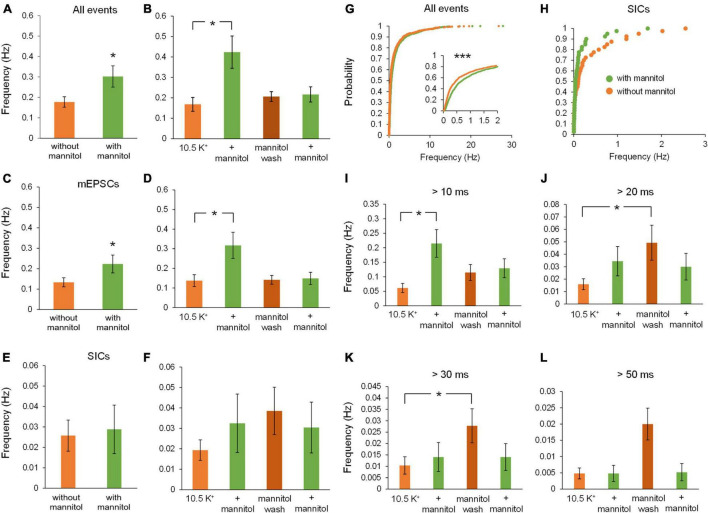
Addition of mannitol increases overall event frequency but frequency of slower events is increased after mannitol removal. The frequency of events was obtained for the combined 20-min applications of each solution, as well as for individual 10-min recording sequences. **(A)** Average event frequency during exposure to ^∧^[K^+^]_*o*_ alone was much lower than during addition of mannitol (*n* = 9). **(B)** Frequency of all events was drastically increased when mannitol was first introduced, but not with the second mannitol application (*n* = 8). **(C)** Average frequency of mEPSCs was higher in mannitol compared to ^∧^[K^+^]_*o*_ alone (*n* = 9), similar to what was observed for all event types. **(D)** Average event frequency sharply increased with the initial addition of mannitol, then returned to near ^∧^[K^+^]_*o*_ levels (*n* = 8), again similar to what was observed for all events pooled together. **(E)** Frequency of SICs was not affected overall by addition of mannitol (*n* = 9). **(F)** Although there was a slight increase in SIC frequency when mannitol was first applied, this change was not statistically significant (*n* = 8). **(G)** Similar to **(A)**, cumulative probability distribution of instantaneous frequency (calculated from inter-event intervals) revealed a greater frequency of mixed events in mannitol compared to ^∧^[K^+^]_*o.*_ (*n* = 1908 events). **(H)** For SICs, a rightward shift in cumulative probability distribution of instantaneous frequency was not significant, likely due to the lower prevalence of these events overall (*n* = 48 events). **(I)** Events with rise time >10 ms occur with the greatest frequency during the first mannitol application (*n* = 9), likely due to “bleeding in” of synaptic events into this pool. **(J)** Frequency of events >20 ms did not increase during mannitol application but instead increased during the mannitol wash period compared to the first ^∧^[K^+^]_*o*_ application (*n* = 9). **(K)** Events with rise time >30 ms follow a similar trend as 20 ms events but with an even more pronounced increase during the mannitol wash period (*n* = 9). **(L)** Events with rise time >50 ms followed the same trends as 20 and 30 ms events, but were too infrequent overall to draw significant conclusions (*n* = 9). *p < 0.05 and ***p < 0.001.

Figure 3 illustrates a representative neuronal recording across alternating periods of ^∧^[K^+^]_*o*_ and hyperosmolar ^∧^[K^+^]_*o.*_ During the first ^∧^[K^+^]_*o*_ application, there was an evident inward shift in the holding current to maintain voltage-clamp at −70 mV, followed by a reduction in the holding current in hyperosmolar ^∧^[K^+^]_*o*_ (baseline 2.5 mM [K^+^]_*o*_, −27.9 ± 4.6 pA; 10.5 mM [K^+^]_*o*_, −147.4 ± 13.6 pA; ^∧^[K^+^]_*o*_ + mannitol, −125.4 ± 11.7 pA; *p* < 0.05) (Figures 3A,B). The second application cycle proceeded much in the same manner as the first, but with a more pronounced occurrence of large SIC-like events upon removal of mannitol (mannitol wash, −156.9 ± 12.9 pA; ^∧^[K^+^]_*o*_ + mannitol 2nd application, −129.9 ± 9.2 pA; *p* < 0.05) (Figures 3A,B). The section of the trace represented in Figure 3C highlights the scale and frequency of large mixed NMDA/AMPA receptor events. Addition of mannitol was characterized by an apparent reduction in slow, large amplitude events (Figure 3D). Overall, application of mannitol resulted in a net increase in total event frequency compared to ^∧^[K^+^]_*o*_ alone (0.18 ± 0.03 Hz in ^∧^[K^+^]_*o*_, 0.30 ± 0.03 Hz in ^∧^[K^+^]_*o*_ + mannitol; *p* < 0.05) (Figure 4A). When mannitol was initially added to the solution, the average frequency of all events increased (^∧^[K^+^]_*o*_, 0.17 ± 0.03 Hz; ^∧^[K^+^]_*o*_ + mannitol, 0.42 ± 0.08; *p* < 0.05), but not in the subsequent 10 min application cycles (Figure 4B). We next analyzed mEPSCs and SICs independently to further examine what types of events underlie this effect.

While frequency of mEPSCs was higher in hyperosmolar conditions overall (Figure 4C), this increase only occurred during the first addition of mannitol (^∧^[K^+^]_*o*_, 0.14 ± 0.03 Hz; ^∧^[K^+^]_*o*_ + mannitol 0.32 ± 0.07 Hz; *p* < 0.05) (Figure 4D). This effect was attributed to mannitol acting as a secretagogue, forcing fusion of synaptic vesicles with the presynaptic terminal upon neuronal shrinking (Kettenmann and Grantyn, 1992). However, the second mannitol application did not evoke as many events as the first mannitol application and was not significantly different compared to ^∧^[K^+^]_*o*_ alone (Figure 4D). This effect may be due to limited availability of docked synaptic vesicles, which may not have had time to restore following the first mannitol application (Marx et al., 2015). Hyperosmotic solutions have been shown to stimulate expenditure of this pool of docked vesicles in a calcium-independent mode of exocytosis (Rosenmund and Stevens, 1996). Unlike effects on mixed AMPA/NMDA receptor mEPSCs, there was no significant effect on frequency of SICs by addition of mannitol compared to ^∧^[K^+^]_*o*_ alone either across all time periods (Figure 4E) or between each 10 min application cycle (*p* = 0.05 by Friedman test) (Figure 4F). Because SIC events are considered to be driven by extrasynaptic and/or non-vesicular sources of glutamate, they are not likely to be affected by a secretagogue which would predominantly target postsynaptic receptors and thereby generate mainly mEPSCs.

Cumulative probability analysis largely corroborated findings on average frequency across cells (*p* < 0.001) (Figure 4G). While there was an apparent increase in the frequency of SICs with initial exposure to mannitol, this result was not statistically significant (Figure 4H). To further isolate effects of cell/ECS volume on neuronal excitability, frequency of events occurring in each condition was analyzed using increasing rise time cutoffs (Figures 4I–L). At a cutoff of 10 ms, mannitol application still resulted in an increased frequency of events compared to the initial period in ^∧^[K^+^]_*o*_ alone (Figure 4I). However, this effect disappeared when comparing events >20 ms. Furthermore, frequency of events >20 ms actually increased during the mannitol wash period after the first mannitol application compared to the initial period in ^∧^[K^+^]_*o*_ alone, during a period of time when the increase in cell volume is greatest (Figures 4J,K). These results are in line with prior experiments showing that SICs are evoked by cellular swelling (Fiacco et al., 2007; Lauderdale et al., 2015). While this trend appeared to extend to events with rise time >50 ms, there were not enough events in this category to draw significant conclusions (*p* = 0.021 by Friedman test; *p* > 0.05 for all *post hoc* comparisons) (Figure 4L).

**FIGURE 5:**
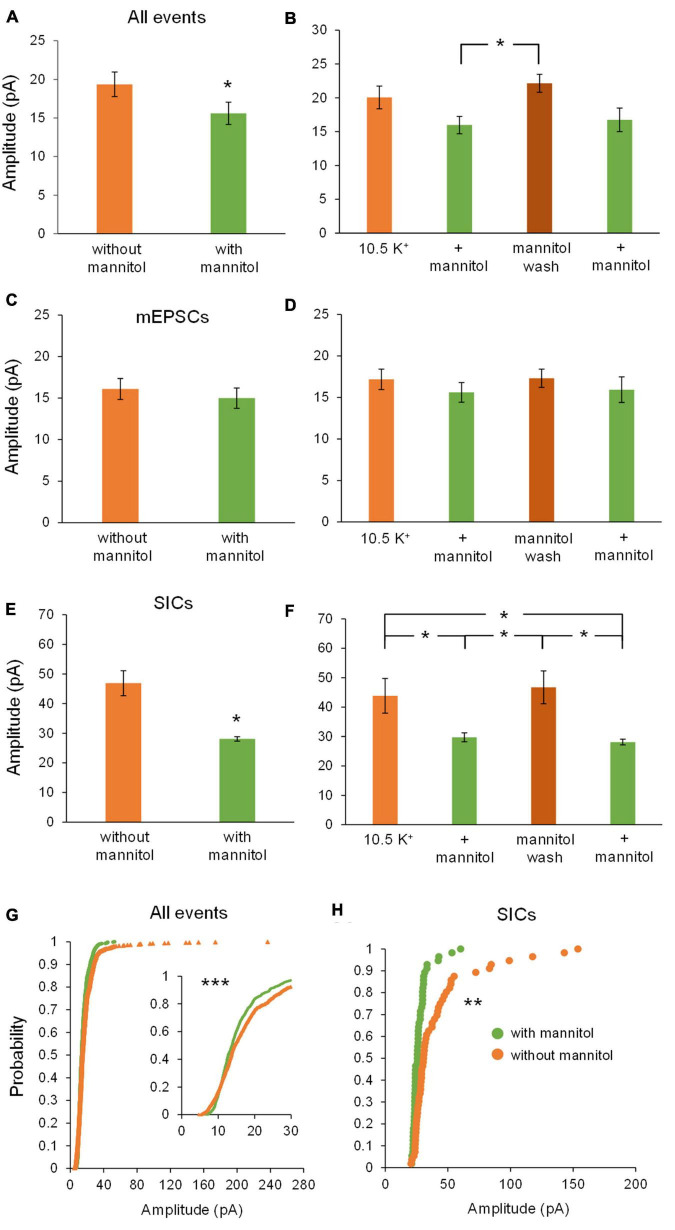
Amplitude of all events and SICs, but not mEPSCs, decrease with addition of mannitol. **(A)** Events occurring across all 20 min in ^∧^[K^+^]_*o*_ had higher amplitudes on average compared to events occurring in ^∧^[K^+^]_*o*_ + mannitol (*n* = 9). **(B)** Amplitudes increased sharply during the mannitol wash period following the first mannitol application (*n* = 8). **(C)** Mannitol had no effect on mEPSC amplitude when combining ^∧^[K^+^]_*o*_ only and + mannitol periods (*n* = 9) or **(D)** when comparing among individual 10 min recording intervals (*n* = 8). **(E)** Amplitude of SICs was significantly diminished in mannitol compared to ^∧^[K^+^]_*o*_ alone (*n* = 8). **(F)** Amplitude of SICs was significantly reduced during the first application of mannitol, but then increased back to ^∧^[K^+^]_*o*_ levels during the mannitol wash period. The second application of mannitol once again significantly reduced SIC amplitude (*n* = 7). **(G)** In the cumulative probability distribution, the leftward shift for events in mannitol reflects a reduced amplitude overall compared to events in ^∧^[K^+^]_*o*_ (*n* = 1962 events). **(H)** Likewise, cumulative probability analysis for SICs revealed lower amplitudes in the + mannitol condition compared to ^∧^[K^+^]_*o*_ alone (*n* = 60 events). *p < 0.05, **p < 0.01, and ***p < 0.001.

We next examined changes in peak event amplitude between ^∧^[K^+^]_*o*_ vs. ^∧^[K^+^]_*o*_ + mannitol conditions. Unlike frequency, event amplitudes were significantly larger during exposure to ^∧^[K^+^]_*o*_ compared to ^∧^[K^+^]_*o*_ + mannitol (Figure 5A). The sharpest increase in amplitude was seen during the mannitol wash period following the first mannitol application (+ mannitol, 15.9 ± 1.3 pA; mannitol wash, 22.1 ± 1.3 pA; *p* < 0.05) (Figure 5B). This effect was also readily apparent when looking at the individual recordings (see Figures 3C,D). Effects on amplitude were event type-specific. For mEPSCs there was no significant effect of mannitol relative to ^∧^[K^+^]_*o*_ alone across all application periods (20 min bins; Figure 5C), or when comparing between individual applications (10 min bins; Figure 5D). For SICs, however, there was a significant reduction in amplitude during mannitol application periods compared to ^∧^[K^+^]_*o*_ alone (Figure 5E). This mannitol-dependent reduction of SICs was also seen when comparing between the first ^∧^[K^+^]_*o*_ to mannitol application periods (^∧^[K^+^]_*o*_, 43.8 ± 5.9 pA; ^∧^[K^+^]_*o*_ + mannitol 1st application, 29.7 ± 1.5 pA; *p* < 0.05), as well as the mannitol wash period relative to either + mannitol condition (mannitol wash, 46.7 ± 5.6 pA; ^∧^[K^+^]_*o*_ + mannitol 2nd application, 28.1 ± 0.1 pA; *p* < 0.05) (Figure 5F). SICs are known to occur with a variety of amplitudes, some near the size of synaptic events and others well over 100 pA (Kovács and Pál, 2017). We observed that larger-amplitude SICs were much more likely to occur while the ECS volume was constricted, decreasing the distance between neuronal and astrocytic membranes. This was clearly apparent when charting cumulative probability for amplitudes of all events combined (*p* < 0.001) as well as for SICs specifically (*p* < 0.01) (Figures 5G,H).

**FIGURE 6:**
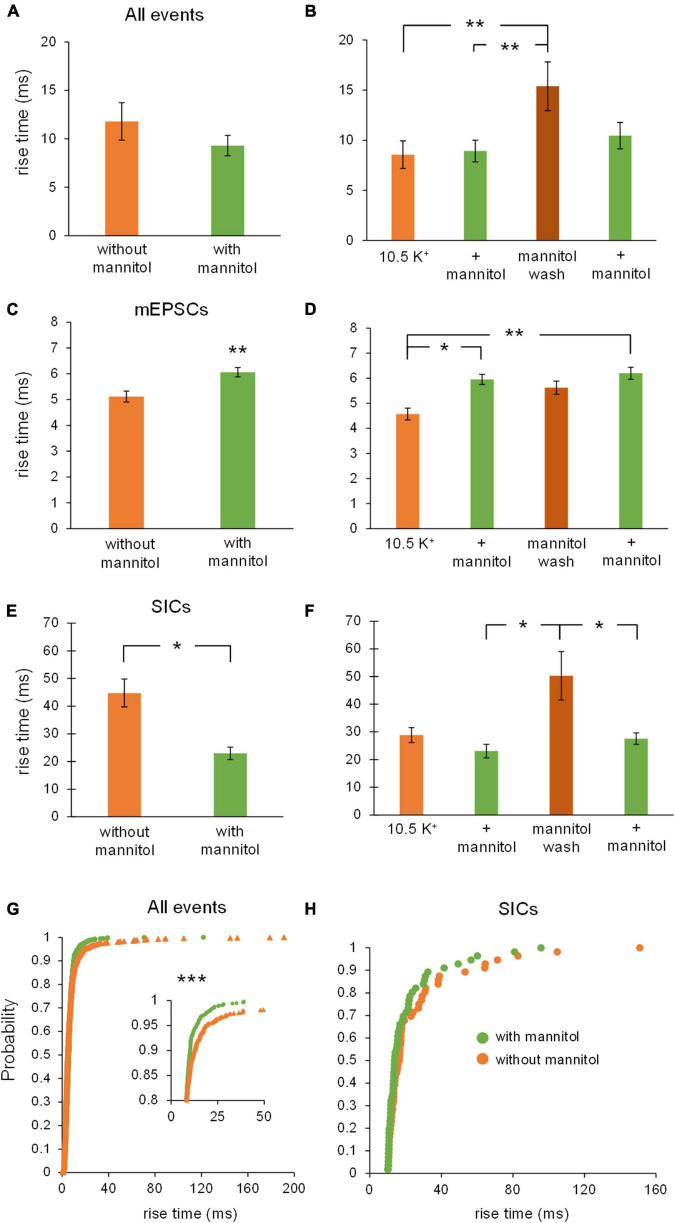
Rise times of mEPSCs and SICs are differentially affected by mannitol. **(A)** Overall, event rise times were slightly but not significantly faster in the presence of mannitol compared to ^∧^[K^+^]_*o*_ alone (*n* = 9). **(B)** For individual 10-min applications, events occurring during the mannitol wash period were much slower than events during the preceding mannitol application period or the initial period in ^∧^[K^+^]_*o*_ (*n* = 8). **(C)** Mixed AMPA/NMDA receptor mEPSCs were significantly slower in ^∧^[K^+^]_*o*_ + mannitol compared to those occurring in ^∧^[K^+^]_*o*_ alone (*n* = 9). **(D)** mEPSCs occurring during both mannitol applications were slower than events occurring during the initial ^∧^[K^+^]_*o*_ application (*n* = 8). **(E)** Unlike mEPSCs, SICs had faster rise times during periods of mannitol exposure compared to periods in ^∧^[K^+^]_*o*_ (*n* = 8). **(F)** The slowest SICs occurred during the mannitol wash period when cells were swelling the most, with faster events occurring during both mannitol applications (*n* = 7). **(G)** Cumulative probability showed a leftward shift for event rise times in mannitol, suggesting that increasing the osmolarity of ^∧^[K^+^]_*o*_ ACSF results in faster rise times overall (*n* = 1962 events). **(H)** Cumulative probability analysis revealed no effect on SIC rise times (*n* = 60 events). *p < 0.05, **p < 0.01, and ***p < 0.001.

Similar effects were observed when examining effects on rise time across varying osmolarity conditions. When comparing all events between ^∧^[K^+^]_*o*_ vs. + mannitol conditions, there was no overall effect on rise time, likely because of the large number of synaptic events included in this pool (Figure 6A). However, rise times were dramatically slower during the mannitol wash period compared to the initial application of ^∧^[K^+^]_*o*_, and to the preceding period in mannitol (^∧^[K^+^]_*o*_, 8.5 ± 1.4 ms; ^∧^[K^+^]_*o*_ + mannitol, 8.9 ± 1.1 ms; mannitol wash, 15.4 ± 2.4 ms; *p* < 0.01) (Figure 6B). Further analysis by event type revealed that mEPSCs and SICs undergo opposite changes in rise time. Rise time of mEPSCs *increased* in the presence of mannitol compared to ^∧^[K^+^]_*o*_ (Figures 6C,D), while rise time of SICs *decreased* (Figure 6E). Specifically, the slowest SICs occurred during the mannitol wash period, while rise times were significantly faster during both applications of mannitol (1st mannitol application, 23.1 ± 2.4 ms; mannitol wash, 50.3 ± 8.8 ms; 2nd mannitol application, 27.6 ± 2.1 ms; *p* < 0.05) (Figure 6F). Cumulative probability analysis showed that rise time was shifted toward faster events during mannitol application compared to ^∧^[K^+^]_*o*_ alone (*p* < 0.001) (Figure 6G). This is likely due to the presence of slower SICs driving the rise time of overall events (compare to Figures 6A,C,E). Cumulative probability analysis for SICs did not indicate a significant effect of mannitol (Figure 6H). However, this may be attributed to the relatively rare occurrence of SICs compared to other event types, leading to a sparser distribution of events.

Taken together, these findings show that when the ECS volume is forced to expand by addition of mannitol, rapid and low amplitude synaptic events occur more frequently due to increased vesicular fusion (Figure 4G), while larger and slower SIC events are diminished (Figures 4J,K). Despite mannitol’s positive modulation of mEPSC activity, expansion of the ECS negatively modulates the occurrence of SICs, which are the most likely to synchronize activity among and between adjacent neurons (Angulo et al., 2004; Fellin et al., 2004; Qiu et al., 2014; Lauderdale et al., 2015). These data indicate that a significant portion of the excitability generated in elevated K^+^ conditions is due to cell swelling and reduced volume of the ECS, and that this activity can be effectively isolated from neuronal quantal vesicular events.

### Block of AMPA Receptors Isolates NMDA Receptor Contribution to Neuronal Excitability in ^∧^[K^+^]_*o*_

**FIGURE 7:**
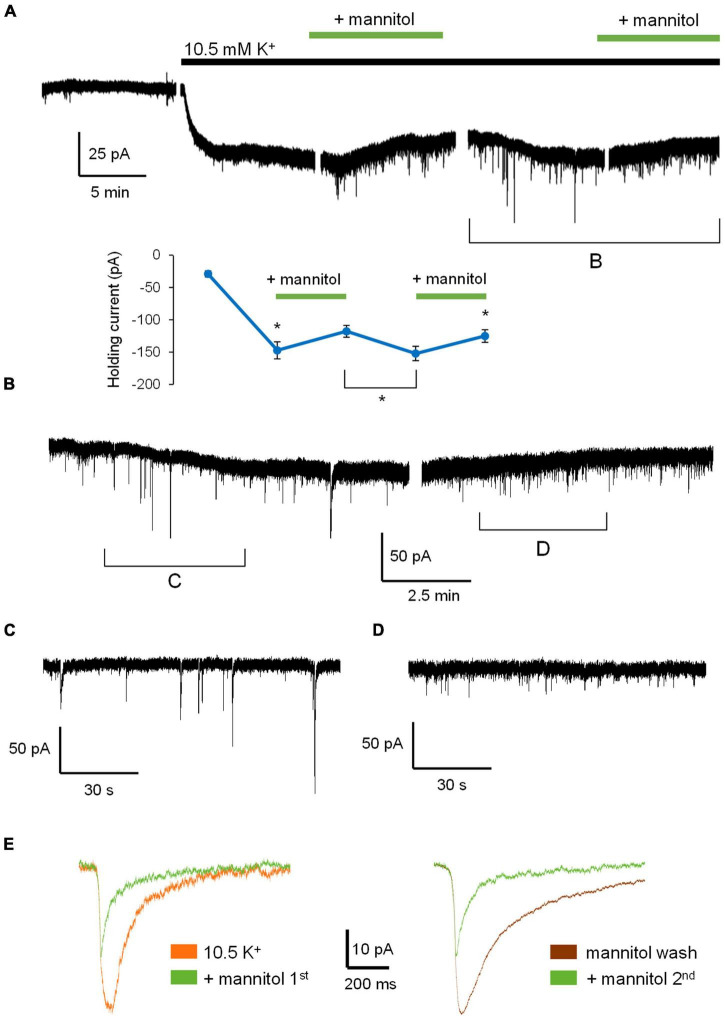
Application of hyperosmolar ^∧^[K^+^]_*o*_ triggers changes in neuron membrane excitability despite block of AMPARs. **(A)** Representative recording of a neuron voltage-clamped at –70 mV in Mg^2+^-free ACSF + 1 μM TTX and 10 μM NBQX. Gaps in recordings coincide with periodic measurement membrane potential and holding current in order to assess patch-clamp quality between alternating 10-min ACSF treatments: control ACSF, ^∧^[K^+^]_*o*_, ^∧^[K^+^]_*o*_ + mannitol, mannitol wash, and ^∧^[K^+^]_*o*_ + mannitol. **(B)** Section of recording showing 10-min mannitol wash and 10-min second application of ^∧^[K^+^]_*o*_ + mannitol. Inset shows the value of the holding current measured at the end of each application period (*n* = 13). **(C)** An ∼ 4-min section of the recording in **(B)**, showing events during the mannitol wash period in ^∧^[K^+^]_*o*_. **(D)** An ∼ 4-min section of the recording in **(B)**, showing events during the second ^∧^[K^+^]_*o*_ + mannitol application. Note the large SICs during the mannitol wash period compared to the smaller mEPSCs present during the second ^∧^[K^+^]_*o*_ + mannitol co-application. **(E)** A comparison of averaged SIC traces from the first application of ^∧^[K^+^]_*o*_ with the first ^∧^[K^+^]_*o*_ + mannitol co-application (left) and between the mannitol wash and the second ^∧^[K^+^]_*o*_ + mannitol co-application (right). *p < 0.05.

Increased prevalence of SICs during hypoosmolar reduction in the volume of the ECS has been tied to activity of NMDA receptors (Lauderdale et al., 2015). Expanding upon this, we sought to isolate the activity of NMDA receptors in ^∧^[K^+^]_*o*_ ACSF through the selective block of AMPA receptors using NBQX. Despite removing contribution from AMPARs to excitatory events, we observed similar effects of mannitol on neuronal excitability in ^∧^[K^+^]_*o*_. Mannitol application appeared to increase the frequency of events overall compared to the initial ^∧^[K^+^]_*o*_ application, while producing a hyperpolarizing shift in the holding current (Figure 7A). Upon mannitol removal, there was a clear increase in the appearance of large NMDA receptor events which again diminished during the second co-application of mannitol in ^∧^[K^+^]_*o*_ (Figures 7A–D). Overlays of averaged SICs across all cells, one comparing the initial application of ^∧^[K^+^]_*o*_ to the first co-application of mannitol, and another comparing the mannitol wash period to the second co-application of mannitol, revealed hyperosmolarity-induced reduction of SIC amplitude and kinetics (Figure 7E). The continued observation of significant effects on SICs in the presence of NBQX suggested that AMPA receptors are not required for the neuronal excitability changes, although they are likely to contribute to mixed events that include both AMPA and NMDA receptor components.

**FIGURE 8:**
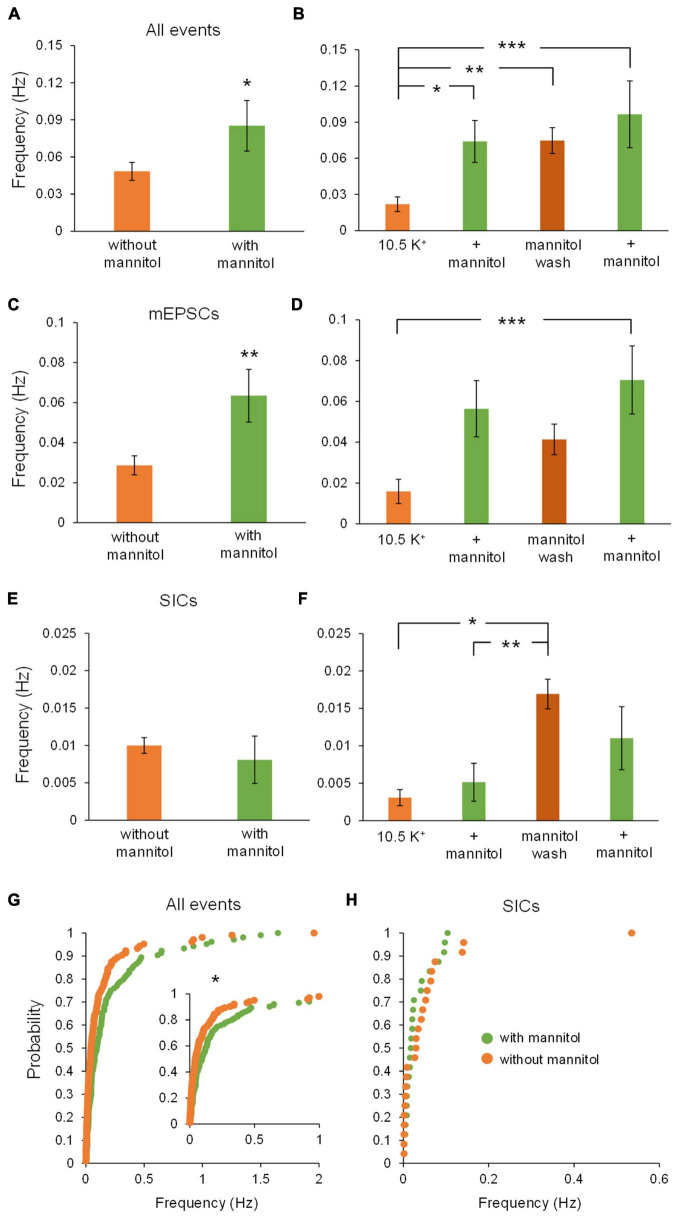
Increased frequency of all events and NMDA receptor mEPSCs during mannitol application, while SIC frequency increases upon mannitol removal. Average frequency of events was calculated for the combined 20-min applications of each solution, as well as for individual 10-min bins. **(A)** Average frequency of all event types was higher in the presence of ^∧^[K^+^]_*o*_ + mannitol than in ^∧^[K^+^]_*o*_ alone (*n* = 13). **(B)** Average event frequency increased during both 10 min applications of mannitol as well as the mannitol wash period (*n* = 13). **(C)** Average frequency of mEPSCs was higher during co-application of ^∧^[K^+^]_*o*_ + mannitol than during application of ^∧^[K^+^]_*o*_ alone (*n* = 13). **(D)** Average frequency of mEPSCs increased during the second co-application of ^∧^[K^+^]_*o*_ + mannitol relative to the initial application of ^∧^[K^+^]_*o*_ alone, with non-significant fluctuations in frequency during the first ^∧^[K^+^]_*o*_ + mannitol co-application and mannitol wash periods (*n* = 13). **(E)** There was no significant difference between the frequency of SICs during ^∧^[K^+^]_*o*_ + mannitol co-application vs. ^∧^[K^+^]_*o*_ alone (*n* = 13). **(F)** Frequency of SICs significantly increased during the mannitol wash period relative to both the initial ^∧^[K^+^]_*o*_ application and the first ^∧^[K^+^]_*o*_ + mannitol co-application (*n* = 13). **(G)** Cumulative probability distribution of instantaneous frequency (calculated from inter-event intervals) showed a greater frequency of events in ^∧^[K^+^]_*o*_ + mannitol compared to ^∧^[K^+^]_*o*_ alone (*n* = 208 events). **(H)** Cumulative probability distribution of instantaneous frequency showed no difference in SIC frequency across experimental conditions (*n* = 48 events). *p < 0.05, **p < 0.01, and ***p < 0.001.

To further elucidate the role of NMDA vs. AMPA receptors in cell-volume regulated excitability changes, we next quantified the frequencies, amplitudes, and rise times of events in the presence of NBQX. As with our previous experiment, we observed a significant increase in event frequency in ^∧^[K^+^]_*o*_ compared to co-application of mannitol (^∧^[K^+^]_*o*_, 0.0483 ± 0.0073 Hz; ^∧^[K^+^]_*o*_ + mannitol, 0.0853 ± 0.0205 Hz; *p* < 0.05) (Figures 8A,G), again pointing to a secretagogue effect of mannitol. However, unlike with mixed AMPA/NMDA receptor events, the frequency of isolated NMDA receptor events remained elevated during the mannitol wash period, likely reflecting the increased frequency of extrasynaptic SICs during cell swelling in ^∧^[K^+^]_*o*_ (Figure 8B). Separate analysis of NMDA receptor-mediated mEPSCs and NMDAR-dependent SICs was instrumental in further segregating secretagogue effects from volume-driven excitability effects. While frequency of mEPSCs was significantly greater in the presence of mannitol compared to ^∧^[K^+^]_*o*_ alone (Figures 8C,D), frequency of SICs was unchanged overall (Figures 8E,H) but was significantly greater during the mannitol wash period compared to the initial ^∧^[K^+^]_*o*_ application and the preceding stretch of recording in mannitol (Figures 8E,F). These findings suggest that SICs specifically are evoked by cell swelling and reduction of the ECS, while NMDA receptor-dependent synaptic events are a separate population less affected by volume changes but rather sensitive to changes in mechanisms regulating vesicular release. These findings support previous assertions that SICs are driven by extrasynaptic NMDA receptors rather than synaptic receptors, which will preferentially bind glutamate released into the synaptic cleft from the presynaptic terminal.

**FIGURE 9:**
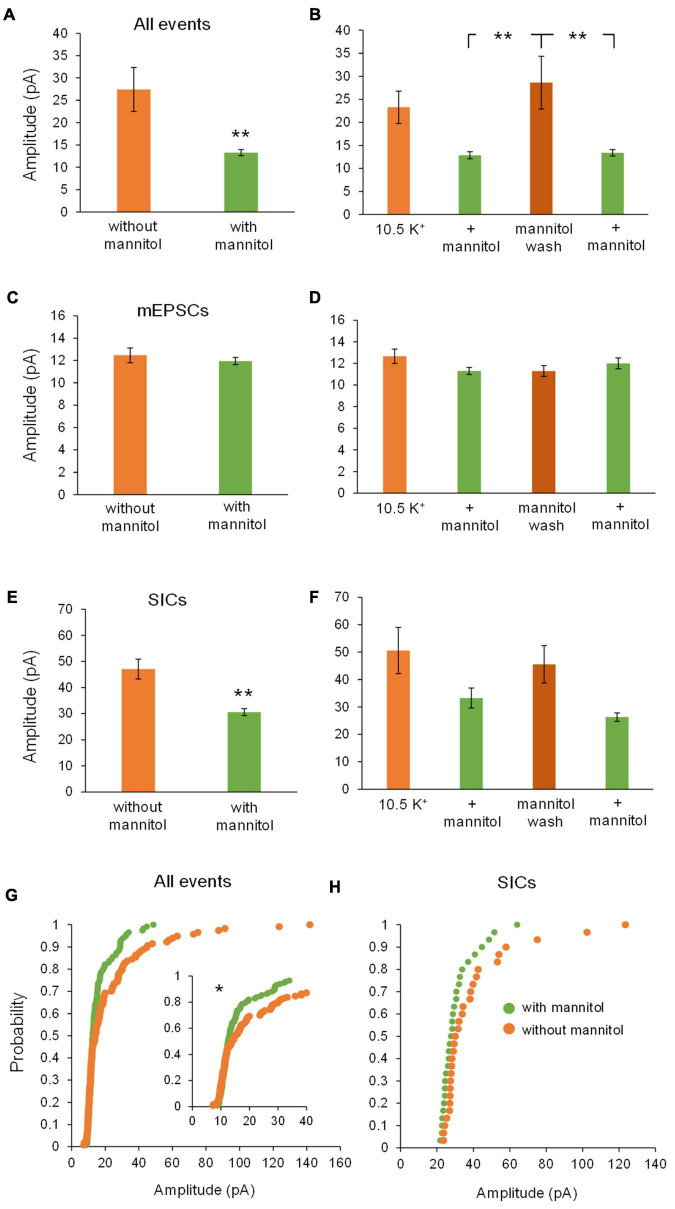
Amplitude of all NMDA receptor events and SIC decreases with the addition of mannitol. **(A)** Average amplitude of all NMDAR-mediated events occurring during co-application of ^∧^[K^+^]_*o*_ + mannitol was lower compared to ^∧^[K^+^]_*o*_ alone (*n* = 13). **(B)** Average amplitude of all events was significantly lower in the presence of mannitol than during the mannitol wash period (*n* = 13). **(C)** There was no significant change in amplitude of mEPSCs with and without mannitol across all time periods (*n* = 13) or **(D)** between separate 10-min time bins (*n* = 11). **(E)** Average amplitude of SICs occurring during co-application of ^∧^[K^+^]_*o*_ + mannitol was lower than in ^∧^[K^+^]_*o*_ alone (*n* = 11). **(F)** Amplitude of SICs was not significantly different in any specific 10 min recording stretch (*n* = 4). The number of statistically usable cells was reduced due to the paucity of SICs in the + mannitol condition, when the extracellular volume expands. **(G)** Cumulative probability analysis reflected the reduced amplitude of all events during co-application of ^∧^[K^+^]_*o*_ + mannitol when compared to application of ^∧^[K^+^]_*o*_ alone (*n* = 234 events). **(H)** Cumulative probability analysis failed to return any significant differences between SICs in the presence of ^∧^[K^+^]_*o*_ + mannitol vs. ^∧^[K^+^]_*o*_ alone (*n* = 60 events). *p < 0.05 and **p < 0.01.

Effects on NMDA receptor current amplitudes were similar to those observed for mixed AMPA/NMDA receptor events and also revealed effects specific to SICs vs. mEPSCs. Once again, there was a significant decrease in event amplitude when assessing all events occurring in ^∧^[K^+^]_*o*_ + mannitol versus ^∧^[K^+^]_*o*_ alone (^∧^[K^+^]_*o*_, 27.4 ± 4.93 pA; ^∧^[K^+^]_*o*_ + mannitol, 13.3 ± 0.68 pA; *p* < 0.01). Amplitudes rebounded significantly during the mannitol wash period when compared to either + mannitol co-application period (Figures 9A,B). There were no significant changes in amplitude of mEPSCs during any recording period (Figures 9C,D). For SICs, there was a significant decrease in amplitude during co-application of ^∧^[K^+^]_*o*_ + mannitol compared to ^∧^[K^+^]_*o*_ alone (Figure 9E), but this effect did not reach significance when comparing individual 10 min recording periods, likely due to the diminished sample size (see statistics section) (*p* > 0.05 for pairwise comparisons after *post hoc* correction) (Figure 9F). This trend was also observed in the cumulative probability analysis, which revealed significantly diminished amplitudes in the + mannitol condition relative to ^∧^[K^+^]_*o*_ alone for all events, but not when SICs were isolated (*p* = 0.1344) (Figures 9G,H). Larger amplitude of events observed during application of ^∧^[K^+^]_*o*_ alone was consistent with the finding that reduction of the ECS promotes SIC activity, while the smaller events observed during co-application of ^∧^[K^+^]_*o*_ and mannitol were consistent with the known secretagogue effect of mannitol (Kettenmann and Grantyn, 1992) in combination with diminished SIC activity when the ECS volume expands.

**FIGURE 10:**
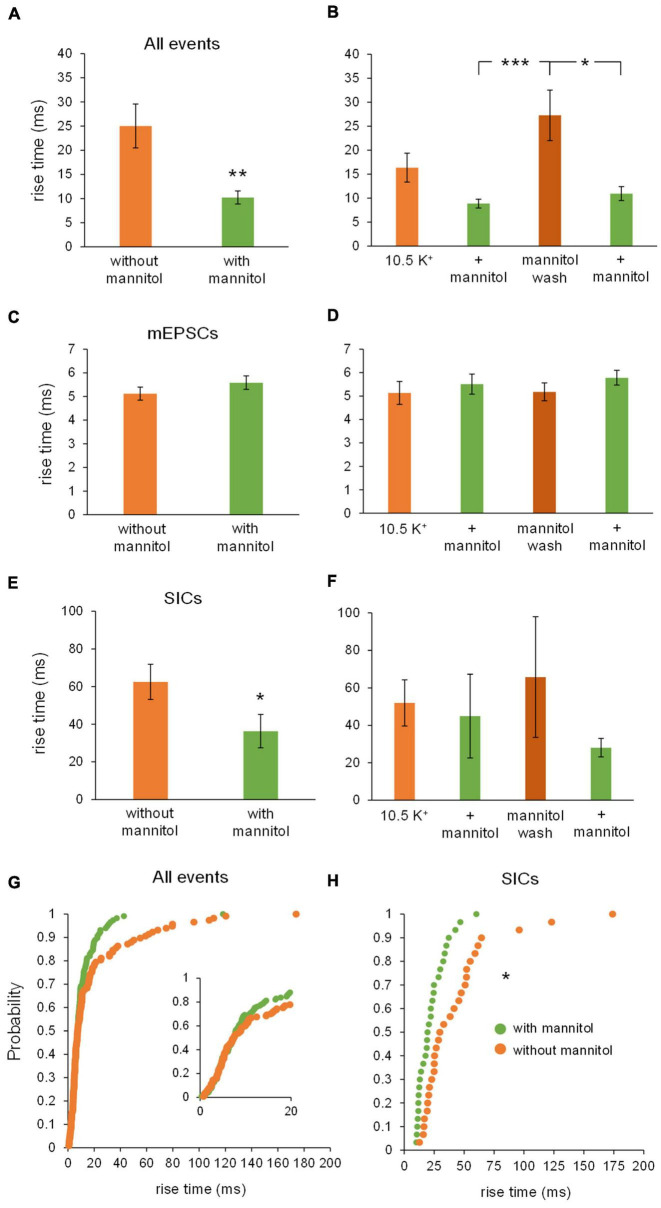
Rise times of all NMDA receptor currents and SICs, but not mEPSCs, become faster in mannitol. **(A)** Rise times for all NMDAR events were significantly faster during co-application of ^∧^[K^+^]_*o*_ + mannitol compared to ^∧^[K^+^]_*o*_ alone (*n* = 13). **(B)** Rise times for all NMDAR events were considerably slower during the mannitol wash period compared to either 10 min period in + mannitol (*n* = 13). **(C)** There were no significant differences in the rise times for NMDAR mEPSCs during ^∧^[K^+^]_*o*_ application with and without mannitol (*n* = 13). **(D)** There was no significant difference in rise times of NMDAR mEPSCs when analyzed in separate 10-min time bins (*n* = 11). **(E)** Rise times for SICs were significantly faster during co-application of ^∧^[K^+^]_*o*_ + mannitol than during ^∧^[K^+^]_*o*_ application alone (*n* = 11). **(F)** Rise times for SICs were not significantly different when compared across individual 10 min recording periods (*n* = 4). The number of statistically usable cells was reduced due to the scarcity of SICs occurring in the + mannitol condition. **(G)** Cumulative probability analysis failed to return any significant difference in rise times of all NMDAR events in ^∧^[K^+^]_*o*_ vs. ^∧^[K^+^]_*o*_ + mannitol (*n* = 234 events). **(H)** Cumulative probability revealed a significant leftward shift toward faster rise times for SICs in mannitol, suggesting that dilating the extracellular space sped up the rate of glutamate diffusion in the ECS (*n* = 60 events). *p < 0.05, **p < 0.01, and ***p < 0.001.

Effects on rise times of isolated NMDA receptor events were similar to effects on amplitude. Co-application of ^∧^[K^+^]_*o*_ + mannitol significantly diminished event rise times compared to ^∧^[K^+^]_*o*_ alone (Figure 10A). Again, effects were greatest during the mannitol wash period relative to the preceding and subsequent recording periods in ^∧^[K^+^]_*o*_ + mannitol (Figure 10B). These differences appeared to be tied to SICs, since there were no significant differences in the rise times of NMDA receptor-dependent mEPSCs (Figures 10C,D). There was a significant decrease in the rise time of SICs in ^∧^[K^+^]_*o*_ + mannitol conditions overall (Figure 10E), with this decrease being responsible for the trend seen in all events, but no significant differences were found between individual sections of recording again due to the overall rarity of SICs in smaller bin sizes (see statistics section; *p* = 0.145 for Freidman test) (Figure 10F). A purely visual inspection of cumulative probability of rise time for all NMDA receptor events suggests that the probability of an increased rise time is higher for ^∧^[K^+^]_*o*_ alone when compared to ^∧^[K^+^]_*o*_ with mannitol, although this cannot be stated definitively (Figure 10G). However, for SICs specifically, despite the overall sparsity of events there was a significant leftward shift in cumulative probability reflecting slower rise times during periods of compression of the ECS (Figure 10H). The slower rise times observed during periods of ^∧^[K^+^]_*o*_-induced swelling can be attributed to the aforementioned increase in SICs when the volume of the ECS is reduced.

### Application of DL-AP5 Reduces Slow Inward Currents and Overall Excitability in ^∧^[K^+^]_*o*_

**FIGURE 11:**
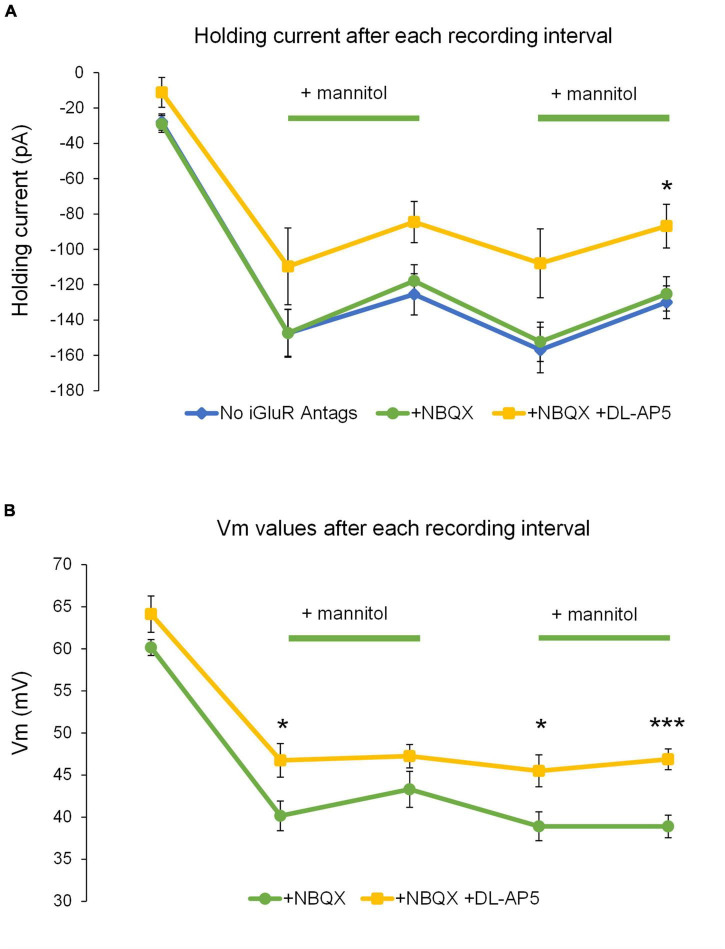
Addition of DL-AP5 attenuates volume-related effects on neuronal excitability. **(A)** Generally, the amount of holding current to maintain voltage-clamp at –70 mV was reduced in ^∧^[K^+^]_*o*_ + mannitol compared to ^∧^[K^+^]_*o*_ alone in all experiments. Holding currents for mixed AMPA + NMDA receptor experiments (blue, *n* = 9) and those isolating NMDA receptor currents (+NBQX) (green, *n* = 13) appeared remarkably similar and had no points during which they were significantly different. Holding currents recorded during the NMDA receptor inhibition experiments (+NBQX/+DL-AP5) were less negative overall and were significantly less negative following the second ^∧^[K^+^]_*o*_ + mannitol co-application period (yellow, *n* = 8). **(B)** Comparison of resting membrane potentials for the NMDA receptor isolation and NMDA receptor inhibition experiments. Shifts in resting membrane potential indicated that cells became depolarized relative to baseline during application of ^∧^[K^+^]_*o*_, while co-application of ^∧^[K^+^]_*o*_ + mannitol triggered slight hyperpolarizing shifts. Resting membrane potentials recorded during the NMDAR inhibition experiments (yellow, *n* = 8) indicated significantly less depolarization compared to the NMDAR isolation experiments following the initial application of ^∧^[K^+^]_*o*_ alone, the mannitol wash period, and the second co-application of ^∧^[K^+^]_*o*_ + mannitol (green, *n* = 13). *p < 0.05 and ***p < 0.001.

**FIGURE 12:**
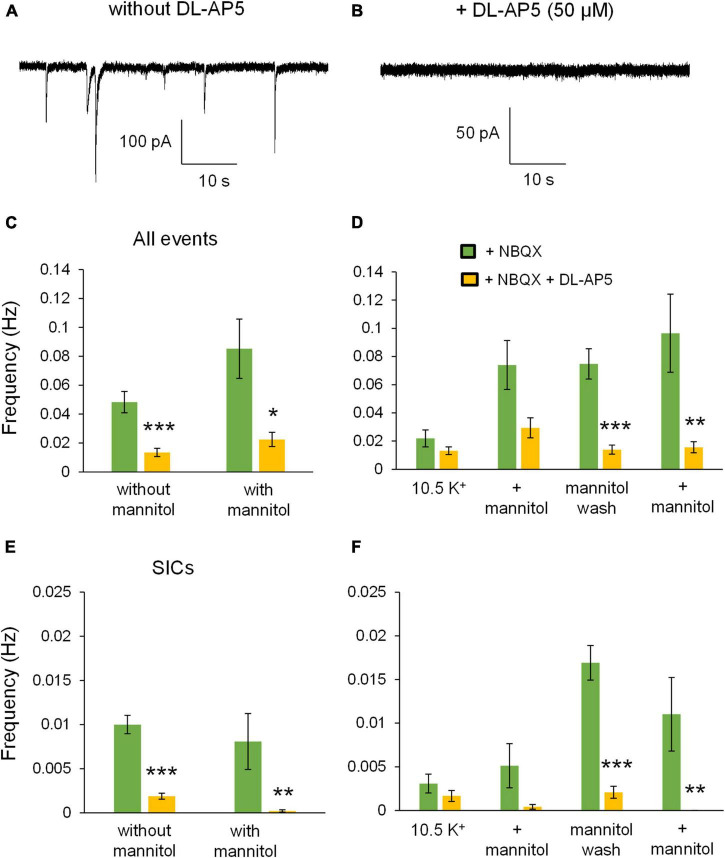
DL-AP5 substantially attenuates NMDA receptor currents during application of ^∧^[K^+^]_*o*_. Approximately 1-min section of recording taken from the mannitol wash period for an experiment conducted in NBQX without DL-AP5 **(A)** compared to the mannitol wash period with 50 μM DL-AP5 **(B)**. Note the number of large SIC-like events in the absence, but not the presence, of DL-AP5. **(C)** Frequency of all NMDA receptor events was significantly lower in DL-AP5 (yellow, *n* = 8) compared to + NBQX alone (green, *n* = 13) during ^∧^[K^+^]_*o*_ application both with and without mannitol. **(D)** When separating events into individual 10-min recording periods, NMDA receptor events were significantly reduced during the mannitol wash period and second ^∧^[K^+^]_*o*_ + mannitol application period. **(E)** DL-AP5 also significantly inhibited the occurrence of SICs either with or without mannitol. **(F)** When grouping frequencies of SICs into 10-min time bins, DL-AP5 significantly inhibited SICs during the mannitol wash period and second ^∧^[K^+^]_*o*_ + mannitol co-application period. SICs were blocked completely during the second ^∧^[K^+^]_*o*_ + mannitol co-application period. *p < 0.05, **p < 0.01, and ***p < 0.001.

To further test the role of NMDARs in neuronal excitability during ECS reduction, experiments in + NBQX were replicated in the presence of the competitive NMDA receptor antagonist 50 μM DL-AP5. Application of DL-AP5 diminished the holding current required to maintain voltage clamp compared to either no iGluR antagonists (mixed NMDA + AMPA receptor activity) or + NBQX (NMDAR current isolation) conditions (Figure 11A). These findings suggest that NMDA receptors significantly contribute to the tonic currents generated in ^∧^[K^+^]_*o*_. DL-AP5 also significantly reduced the depolarizing shift in membrane potential (Figure 11B), suggesting that NMDA receptors also contribute to the overall amount of neuronal depolarization in ^∧^[K^+^]_*o*_. Application of DL-AP5 also significantly reduced phasic NMDA receptor events including mEPSCs and SICs (Figure 12). DL-AP5 almost completely abolished NMDA receptor currents occurring either in the absence or presence of mannitol (Figures 12A–F), but most notably when comparing the mannitol wash period, when most SICs are typically occurring as the ECS dramatically shrinks during cell swelling in ^∧^[K^+^]_*o*_ (Figures 12A,B,D,F). Effects of DL-AP5 were even more pronounced when analyzing SICs independently. Perhaps most strikingly, SICs were completely abolished by DL-AP5 during the second ^∧^[K^+^]_*o*_ + mannitol co-application period (-AP5, 0.0110 ± 0.0042 Hz; + AP5, 0.0 ± 0.0 Hz; *p* < 0.01) (Figures 12E,F). Because DL-AP5 contains a racemic mixture of D- and L-enantiomers, it is likely that these effects would be even more pronounced when using either a higher concentration of the antagonist or if only the active enantiomer D-AP5 were used at this concentration. Overall, these differences illustrate the considerable role that NMDA receptors play in the generation of SICs and in the modulation of neuronal excitability during astrocytic swelling.

## Discussion

In this study, we explored the potential impact of cellular volume changes on elevated neuronal excitability occurring in (patho)physiological increases in [K^+^]_*o*_. Application of 10.5 mM [K^+^]_*o*_ in the presence of tetrodotoxin to prevent neuronal firing elevated CA1 neuronal excitability in the form of mixed AMPA/NMDA receptor mediated mEPSCs and large NMDA receptor-dependent slow inward currents (SICs). Neuronal excitability changes correlated with rapid (within 1 min) astrocyte swelling, while neuronal volume remained relatively constant in ^∧^[K^+^]_*o*_. In order to assess astrocyte swelling effects on recorded excitability changes, hyperosmolar ACSF by addition of mannitol was applied to force astrocyte shrinking in the continued presence of ^∧^[K^+^]_*o*_. Although neuronal volume was also reduced in these conditions, astrocyte swelling in ^∧^[K^+^]_*o*_ was reversed by hyperosmolar ACSF and overall astrocyte volume reduction was approximately two-fold that of neurons. Cellular volume reduction was accompanied by a hyperpolarizing shift in the holding current during whole-cell voltage clamp of CA1 pyramidal neurons, and selectively abolished larger excitatory currents with slow kinetics while having the opposite effect on smaller, faster events. Large SICs returned upon washout of mannitol, confirming the importance of cell swelling for the occurrence of these events. Similar to previous studies using hypoosmolar ACSF to swell cells, SICs generated in ^∧^[K^+^]_*o*_ were significantly attenuated by the NMDA receptor antagonist DL-AP5. Our data suggest that rapid astrocyte swelling contributes to neuronal excitability changes occurring in ^∧^[K^+^]_*o*_ through the generation of large, slow excitatory currents that are mainly driven by activation of NMDA receptors.

Based on previous studies that demonstrated significant and selective astrocyte swelling in ^∧^[K^+^]_*o*_ (Walch et al., 2020) and that cell swelling induced by hypoosmolarity generates SICs (Lauderdale et al., 2015; Yang et al., 2019), we attempted to isolate the astrocyte swelling effects on neuronal excitability increases occurring in ^∧^[K^+^]_*o*_. Forced cellular shrinking in ^∧^[K^+^]_*o*_ by addition of mannitol had a biased effect on large amplitude, slow events over smaller, faster mEPSCs. Miniature EPSCs, whether mixed AMPA/NMDA receptor-mediated mEPSCs or NMDA mEPSCs isolated in the presence of NBQX, actually significantly increased in frequency in the presence of mannitol. This effect is likely due to mannitol acting as a secretagogue, which has been well-documented. Although the mechanism has not been clearly defined, it is thought that hyperosmolar-induced shrinking of neuronal presynaptic terminals evokes calcium-independent exocytosis through forced fusion of synaptic vesicles with the presynaptic membrane (Capogna et al., 1996; Rosenmund and Stevens, 1996; Waseem et al., 2008). Upon removal of mannitol, occurrence of mEPSCs significantly diminished, while slow inward currents dramatically increased, often with much greater frequency and amplitude compared to the initial ^∧^[K^+^]_*o*_ application. The increased prevalence of SICs may be attributable to the nearly two-fold increase in astrocyte swelling upon mannitol removal compared to the initial ^∧^[K^+^]_*o*_ application. We suggest that this rebound change in astrocyte volume may be due to continued pumping of extracellular K^+^ into the astrocytes by the unique astrocyte Na^+^/K^+^ ATPase (Stoica et al., 2017; Walch et al., 2020), despite forced volume reduction by solution hyperosmolarity. Re-application of mannitol again selectively abolished large amplitude, slower events including SICs, while again causing an uptick in miniature synaptic events. This inverse relationship between the generation of mEPSCs vs. SICs hints at a dual action of mannitol, increasing fast synaptic transmission while simultaneously inhibiting slower, volume mediated transmission by dilating the extracellular space.

The distinction between the occurrence of mEPSCs vs. SICs in our study provides further insight into synaptic vs. extrasynaptic activity. SICs have long been considered to be driven by extrasynaptic NMDARs, but the evidence for this has been largely anecdotal. SICs were originally thought to arise from non-synaptic sources of glutamate, specifically, glutamate released by astrocytes. The mechanism was initially ascribed to astrocyte Ca^2+^ elevations (Angulo et al., 2004; Fellin et al., 2004) but astrocyte swelling was also considered to be a critical factor (Kozlov et al., 2006), and SICs were later shown to be strongly evoked in conditions of cell swelling (Fiacco et al., 2007; Lauderdale et al., 2015; Yang et al., 2019). This fit with previous evidence of significant release of glutamate from astrocytes *in vitro* upon application of hypoosmolar solutions to induce swelling (Mongin and Kimelberg, 2002; Kimelberg, 2005; Abdullaev et al., 2006). Based on the location of astrocyte processes relative to synapses, and more diffuse glutamate release from astrocytes relative to neuronal presynaptic terminals, it was logical to predict that astrocytically derived glutamate would preferentially target high affinity extrasynaptic NMDA receptors as opposed to receptors located in the postsynaptic density. This concept gained further traction from early studies suggesting that NR2B subunit-containing NMDA receptors were exclusively extrasynaptic, which subsequent work largely debunked (Rauner and Kohr, 2011; Tovar et al., 2013). Therefore, evidence supporting a role for extrasynaptic NMDARs in the generation of SICs has not been substantiated. In the current study, the inverse relationship between mEPSCs and SICs suggests that these events arise from different receptor populations. While mEPSCs were strongly affected by mannitol’s secretagogue effects, significantly increasing in frequency, SICs were significantly more likely to occur during the mannitol wash period, especially when isolated from fast AMPA receptor events. This supports the idea that the receptors generating these events are located outside the synapse and therefore less affected by miniature vesicular release events. Unlike the closely apposed pre- and post-synaptic membranes (∼24 nm apart) (Peters, 1991; Zuber et al., 2005; Kinney et al., 2013), extrasynaptic iGluRs, which are predominantly NMDARs, may be scattered on other parts of the neuron (Benke et al., 1993; Hardingham and Bading, 2010) and do not have privileged access to presynaptic glutamate (Köhr, 2006). Here, glutamate stimulation likely occurs in a wave-like manner, hitting some NMDARs earlier than others, thus prolonging event duration. Extrasynaptic NMDA receptor events will become even slower during compression of the ECS due to cell swelling, increasing ECS tortuosity and slowing diffusion. Buffering of glutamate by astrocyte glutamate transporters (Tzingounis and Wadiche, 2007) could also add to slower diffusion and action of glutamate onto extrasynaptic receptors. Finding the source of this extracellular glutamate is therefore of high priority and significance.

The mechanism(s) underlying neuronal excitability increases driven by cell swelling remain to be clearly defined. Astrocyte swelling can cause the opening of volume regulated anion channels (VRAC), which are permeable to anions and other osmolytes including glutamate (Kimelberg et al., 1990; Rutledge and Kimelberg, 1996, Mongin and Kimelberg, 2002; Yang et al., 2019). Astrocyte glutamate release through VRAC could excite local neurons through NMDA receptor activation. VRAC have been fervently studied in recent years, and it was previously found that a leucine-rich repeat containing protein 8A (LRRC8A) is the necessary subunit for VRAC-mediated release of glutamate (Hyzinski-García et al., 2014). This makes LRRC8A an interesting target in future studies to determine the contribution of VRAC to neuronal excitability increases in ^∧^[K^+^]_*o*_. Aside from glutamate release via VRAC, it is also possible that astrocyte swelling simply shrinks the extracellular space, elevating ambient glutamate concentrations sufficiently to activate high-affinity extrasynaptic NMDA receptors. This mechanism alludes to the concept of “volume transmission,” which describes intercellular communication facilitated by diffusion of substances through the extracellular space rather than “wired transmission” pathways like the synaptic cleft (Bach-y-Rita, 1993; Agnati et al., 1995). Slow, extrasynaptic diffusion of glutamate is likely a key factor contributing to the kinetics of SICs (Lozovaya et al., 2004). Both of these mechanisms may contribute significantly to the neuronal excitability increases driven by extracellular volume reduction in elevated [K^+^]_*o*_, making them intriguing subjects for future study.

The important role of NMDARs in volume-driven excitability effects was demonstrated by the continued presence of SICs in solutions containing NBQX, which blocks AMPA receptor-dependent activity, and the near total abolition of SICs by DL-AP5, a competitive NMDA receptor antagonist. Addition of DL-AP5 to solutions containing ^∧^[K^+^]_*o*_ led to consistently less excitable neurons, reducing the holding current while hyperpolarizing the membrane potential. Mannitol’s secretagogue effect, however profound it might be, is ultimately insufficient to facilitate the initiation of SICs, and would not likely lead to increased susceptibility to epileptiform activity. This is consistent with numerous studies on the role of mannitol in suppressing neuronal evoked potentials (Ballyk et al., 1991; Saly and Andrew, 1993; Azouz et al., 1997), generation of hypersynchronous neuronal discharges in brain slices (Traynelis and Dingledine, 1989; Dudek et al., 1990; Roper et al., 1992; Kilb et al., 2006), or epileptiform activity in humans with epilepsy (Haglund and Hochman, 2005). Traynelis and Dingledine showed that epileptiform bursting after prolonged exposure to 8.5 mM [K^+^]_*o*_ was NMDA receptor-dependent and required reduction of the extracellular space (Traynelis and Dingledine, 1988). Application of mannitol or sucrose—another known secretagogue—completely blocked NMDA receptor-dependent, spontaneously recurring seizures (Traynelis and Dingledine, 1989). Remarkably, Haglund and Hochman (2005) found in human patients suffering from neocortical and mesial temporal lobe epilepsy that administration of mannitol significantly suppressed spontaneous epileptic spikes and electrical stimulation-evoked epileptiform discharges in all subjects, completely blocking all epileptiform activity in some patients without suppressing normal EEG activity. Even though it is likely that mannitol and sucrose are increasing vesicular fusion in neurons, seizures stop due to cell volume reduction and the marked dilation of the extracellular space. This points to the importance of cell swelling and extracellular space volume regulation as key mediators of neuronal network excitability, arguably playing a far more critical role in hyperexcitable circuits than secretagogue-mediated vesicular release of glutamate. The presence of SICs during cell swelling and reduction of the ECS in ^∧^[K^+^]_*o*_ conditions is particularly intriguing. SICs are a hallmark feature of tissue-swelling induced excitability (Lauderdale et al., 2015; Yang et al., 2019) and their appearance has also been associated with increased neuronal synchrony (Angulo et al., 2004; Fellin et al., 2004), a known characteristic of seizures. The generation of SICs may not be an explicit requirement for epileptiform activity (Fellin et al., 2006), but their presence may serve as a useful diagnostic. Thus, understanding the mechanisms that lead to the generation of SICs may provide a stepping stone toward preventative strategies to treat epilepsy. In the future, it would be prudent to continue to define astrocyte glutamate releasing pathways and search for means to selectively manipulate astrocyte volume bidirectionally in order to isolate astrocyte volume contributions to changes in neuronal and brain tissue excitability.

## Data Availability Statement

The raw data supporting the conclusions of this article will be made available by the authors, without undue reservation.

## Ethics Statement

The animal study was reviewed and approved by the Institutional Animal Care and Use Committee at the University of California, Riverside.

## Author Contributions

EW and TF designed the experiments. AB, EW, and TM performed the experiments. SS, AB, and EW analyzed the data. AL assisted with cell volume measurements. VB assisted with data analysis. EW, AB, SS, and TF wrote the manuscript. TF revised and edited the manuscript. TF, AB, and SS contributed to the finalization and approved the content of the manuscript. All authors contributed to the article and approved the submitted version.

## Conflict of Interest

The authors declare that the research was conducted in the absence of any commercial or financial relationships that could be construed as a potential conflict of interest.

## Publisher’s Note

All claims expressed in this article are solely those of the authors and do not necessarily represent those of their affiliated organizations, or those of the publisher, the editors and the reviewers. Any product that may be evaluated in this article, or claim that may be made by its manufacturer, is not guaranteed or endorsed by the publisher.
